# The Pocket Pharmacy

**Published:** 1892-08

**Authors:** 


THE POCKET PHARMACY, WITH THERAPEUTIC INDEX.—By John Aulde, M. D. Published by D. Appleton & Co., New York.

THIS little book comes to us in nice dress and convenient size; well printed upon good paper.

The author takes up some two dozen different medicines and two or three compounds, and endeavors to give the various pathological conditions, for which the remedies are suited; advocating their use in small and oft repeated doses. The author expresses the hope that the work may be especially useful to the recent graduate, supposed to be "intellectually rich, but technically poor." We fear that the graduate will close the book with the belief that almost any one remedy treated by the author will be ample for the needs of an ordinary practitioner, but he will be unable to decide which one to select, they are all so good.

When we turn to calcium-sulphide and find it recommended for sixty-four different diseases, among them amenorrhœa, deafness, glanders, pertussis, phthisis, pneumonia, sterility, syphilis, tooth-ache and typhoid fever, and close by, find our old friend *calomel* recommended for only thirteen different affections, we are led to believe that an invidious distinction has been made in favor of the calcium sulphide.

